# Applying habitat and population‐density models to land‐cover time series to inform IUCN Red List assessments

**DOI:** 10.1111/cobi.13279

**Published:** 2019-02-25

**Authors:** Luca Santini, Stuart H. M. Butchart, Carlo Rondinini, Ana Benítez‐López, Jelle P. Hilbers, Aafke M. Schipper, Mirza Cengic, Joseph A. Tobias, Mark A. J. Huijbregts

**Affiliations:** ^1^ Department of Environmental Science, Institute for Wetland and Water Research, Faculty of Science Radboud University P.O. Box 9010 NL‐6500 GL Nijmegen The Netherlands; ^2^ BirdLife international David Attenborough Building, Pembroke Street Cambridge CB23QZ U.K.; ^3^ Department of Zoology University of Cambridge Downing Street Cambridge CB23EJ U.K.; ^4^ Department of Biology and Biotechnologies Sapienza Università di Roma Viale dell'Università 32 00185 Rome Italy; ^5^ PBL Netherlands Environmental Assessment Agency P.O. Box 30314 2500 GH The Hague The Netherlands; ^6^ Department of Life Sciences Imperial College London Silwood Park, Buckhurst Road Ascot Berkshire SL5 7PY U.K.

**Keywords:** birds, conservation, data deficient species, extinction risk, mammals, remote sensing, aves, conservación, especies con deficiencia de datos, mamíferos, riesgo de extinción, teledetección, 鸟类, 保护, 数据缺乏的物种, 灭绝风险, 遥感, 哺乳动物

## Abstract

The IUCN (International Union for Conservation of Nature) Red List categories and criteria are the most widely used framework for assessing the relative extinction risk of species. The criteria are based on quantitative thresholds relating to the size, trends, and structure of species’ distributions and populations. However, data on these parameters are sparse and uncertain for many species and unavailable for others, potentially leading to their misclassification or classification as data deficient. We devised an approach that combines data on land‐cover change, species‐specific habitat preferences, population abundance, and dispersal distance to estimate key parameters (extent of occurrence, maximum area of occupancy, population size and trend, and degree of fragmentation) and hence predict IUCN Red List categories for species. We applied our approach to nonpelagic birds and terrestrial mammals globally (∼15,000 species). The predicted categories were fairly consistent with published IUCN Red List assessments, but more optimistic overall. We predicted 4.2% of species (467 birds and 143 mammals) to be more threatened than currently assessed and 20.2% of data deficient species (10 birds and 114 mammals) to be at risk of extinction. Incorporating the habitat fragmentation subcriterion reduced these predictions 1.5–2.3% and 6.4–14.9% (depending on the quantitative definition of fragmentation) for threatened and data deficient species, respectively, highlighting the need for improved guidance for IUCN Red List assessors on the application of this aspect of the IUCN Red List criteria. Our approach complements traditional methods of estimating parameters for IUCN Red List assessments. Furthermore, it readily provides an early‐warning system to identify species potentially warranting changes in their extinction‐risk category based on periodic updates of land‐cover information. Given our method relies on optimistic assumptions about species distribution and abundance, all species predicted to be more at risk than currently evaluated should be prioritized for reassessment.

## Abstract 2

*Article impact statement*: Automatic screening to prioritize species reassessments optimizes the use of resources and keeps the IUCN Red List up to date.

## Abstract (es)

Aplicación de Modelos de Hábitat y de Densidad Poblacional a Series de Tiempo de la Cobertura del Suelo para Informar las Valoraciones de la Lista Roja de la UICN

**Resumen**

Las categorías y los criterios de la Lista Roja de la UICN (Unión Internacional para la Conservación de la Naturaleza) son el marco de referencia utilizado con mayor frecuencia para valorar el riesgo de extinción relativo de las especies. Los criterios se basan en umbrales cuantitativos relacionados con el tamaño, las tendencias y la estructura de la distribución y las poblaciones de las especies. Sin embargo, los datos sobre estos parámetros son escasos e inciertos para muchas especies y para otras no se encuentran disponibles, lo puede resultar en una clasificación errónea o en que se las clasifique como una especie con deficiencia de datos. Hemos diseñado una estrategia que combina datos sobre el cambio en la cobertura del suelo, las preferencias de hábitat específicas por especie, la abundancia poblacional, y la distancia de dispersión para estimar los parámetros más importantes (extensión de la presencia, área máxima de ocupación, tamaño poblacional, y grado y tendencia de la fragmentación) y así predecir las categorías de la Lista Roja de la UICN para cada especie. Hemos aplicado nuestra estrategia a las aves no pelágicas y a los mamíferos terrestres de todo el mundo (∼15,000 especies). Las categorías pronosticadas fueron bastante consecuentes con las valoraciones publicadas por la Lista Roja de la UICN, aunque en general fueron más optimistas. Pronosticamos que el 4.2% de las especies (467 aves y 143 mamíferos) se encuentran más amenazadas que su valoración actual y el 20.2% de las especies con deficiencia de datos (10 aves y 114 mamíferos) se encuentran en riesgo de extinción. La incorporación del sub‐criterio de fragmentación del hábitat redujo estas predicciones en un 1.5 – 2.3% y 6.4 – 14.9% (dependiendo de la definición cuantitativa de la fragmentación) para las especies amenazadas y las que tienen deficiencia de datos, respectivamente, lo que resalta la necesidad de mejorar la aplicación de este sub‐criterio por parte de los asesores de la Lista Roja de la UICN. Nuestra estrategia complementa los métodos tradicionales de estimación de parámetros para las valoraciones de la Lista Roja. Además, proporciona un sistema inmediato de alerta temprana basado en actualizaciones periódicas de la información sobre la cobertura del suelo que permite identificar a las especies que, potencialmente, merezcan un cambio en su categoría de riesgo de extinción. Nuestro método está basado en suposiciones optimistas sobre la distribución y la abundancia de las especies, por lo tanto todas las especies que predecimos que tienen una mayor categoría de riesgo que la que reconoce la evaluación actual deberían ser priorizadas para su revaloración.

## 摘要

世界自然保护联盟 (International Union for Conservation of Nature, IUCN) 红色名录濒危等级和评估标准是物种相对灭绝风险评估中应用得最为广泛的一套框架。这些标准的制定依据与物种分布和种群的大小、趋势和结构有关的定量阈值。然而, 许多物种这些参数的数据稀缺、具有不确定性, 甚至一些物种的数据难以获得和利用, 这可能导致它们被错误地划分濒危等级, 或被归类为“数据缺乏”。我们设计了一种方法, 结合土地覆盖变化、物种特异的生境偏好、种群丰度和扩散距离的数据, 来估计物种的关键参数 (出现范围、最大占有面积、种群大小和趋势、破碎化程度), 从而预测它们的 IUCN 红色名录濒危等级。我们应用这个方法预测了全球非远洋鸟类和陆地哺乳动物 (约 15,000 种) 的濒危等级。预测的濒危等级与 IUCN 红色名录评估结果基本一致, 但总体上更为乐观。预测结果中, 4.2% 的物种 (467 种鸟类和 143 种哺乳动物) 比目前的评估情况更濒危, 且数据缺乏的物种中 20.2% (10 种鸟类和 114 种哺乳动物) 面临灭绝风险。进一步整合生境破碎化的子标准后, 受胁迫物种及数据缺乏物种分别减少了 1.5‐2.3% 和 6.4‐14.9% (取决于对破碎化的定量标准), 这强调了需要更好地指导 IUCN 红色名录评估者对评估标准中相关指标的应用。我们的方法是对红色名录评估中传统的参数估计方法的补充。此外, 它还提供了一个早期预警系统, 可以基于定期更新的土地覆盖数据, 来确定濒危等级可能发生变化的物种。鉴于这一方法依赖于对物种分布和丰度的乐观假设, 所有在预测中比目前评估的濒危风险更大的物种都应该优先进行重新评估。 **【翻译: 胡怡思; 审校: 聂永刚】**

## Introduction

The International Union for Conservation of Nature (IUCN) Red List of Threatened Species is the most authoritative and widely used framework for assessment of extinction risk of species (Rodrigues et al. 2006; IUCN 2017a). Species are assessed using 5 criteria with quantitative thresholds relating to the size, trends, and structure of species’ distributions and populations (Mace et al. 1992; IUCN 2017b). The assessments result in species being listed under 1 of 7 categories, from least concern (LC) to extinct (or data deficient if insufficient information is available to apply the criteria). The IUCN Red List now covers >90,000 species, and a key challenge is to reassess the status of a large proportion of these species periodically and consistently with up‐to‐date data to identify conservation priorities. Reassessments currently rely on information from published and unpublished sources and expert knowledge, but collating relevant data from the literature and from experts for hundreds or thousands of species across wide geographic areas can render the process slow and costly (Rondinini et al. 2014). To increase efficiency, a more systematic, quantitative, and comprehensive approach is needed to support and complement the painstaking work of IUCN Red List assessors.

Assessing species extinction risk requires intense and regular data gathering from all available sources. Although data would ideally be scaled up from data collected on the ground (Pereira et al. 2013), such measurements are relatively costly and time consuming. Furthermore, in situ observations are typically biased geographically and taxonomically due to a number of factors, such as the availability of research funding, emphasis on charismatic species, location of research institutions and researchers, security issues, and accessibility (Wilson et al. 2007; Boakes et al. 2010; Fleming & Bateman 2016). Thus, there is increasing need for new technology, models, and data sets to update, improve, and increase the consistency of assessments for large numbers of species.

The main drivers of biodiversity loss today are overexploitation and habitat loss (Hoffmann et al. 2010; Joppa et al. 2016). Overexploitation is challenging to model in a predictive framework (Benítez‐López et al. 2017), but habitat loss can be inferred indirectly with remote‐sensing data and particularly land‐cover data (Pettorelli et al. 2014). Land‐cover change influences the availability of suitable habitat and, consequently, the potential population size of species. Within the Climate Change Initiative (CCI) of the European Space Agency (ESA), the CCI Land Cover partnership recently released an annual global land‐cover time series covering 24 years from 1992 to 2015 at a resolution of 10 arc‐seconds (∼300 m) (ESA 2017). Further, land‐cover maps from 2016 to 2019 are now being developed in the framework of the Copernicus Climate Change Service (C3S 312b‐lot5) (C. Lamarche, personal communication). These data sets provide an unprecedented opportunity to quantify the effect of land‐cover change on species’ habitat distribution and fragmentation in the recent past.

Land‐cover change data and information on species’ habitat preferences can be coupled to assess how land‐cover change alters the extent of suitable habitat of species and influences their risk of extinction under the IUCN Red List criteria (e.g., Rondinini et al. 2011; Bird et al. 2012; Visconti et al. 2016). For example, recent remotely sensed images of forest cover have been used to assess extinction risk and its recent changes for forest‐dependent species (Buchanan et al. 2008; Tracewski et al. 2016). Such studies typically focus on criteria A (reduction in population size) and B (small and fragmented or declining range) (e.g., Tracewski et al. 2016), whereas few have considered criterion C1 (small and declining population) (e.g. Buchanan et al. 2008) or D1(very small population) (e.g., Visconti et al. 2016).

Here we demonstrated for all nonpelagic birds (10,378 species) and terrestrial (i.e., nonmarine) mammals (4,835 species) how IUCN Red List criteria can be informed by coupling land‐cover time series, species’ habitat preferences, and statistical predictions of species population density and dispersal distance. We used maps of species’ distributions and information on species’ habitat preferences from the IUCN Red List and land‐cover time series data from the ESA to estimate species’ potential distributions and change in these over time. We then estimated the potential population size in suitable habitat and the level of population fragmentation following IUCN Red List guidance. We also assessed the extinction risk of species under 6 IUCN Red List criteria (A2, B1, B2, C1, D1, and D2).

## Methods

### Red List Criteria

For the IUCN Red List, species are assessed against all criteria for which suitable data are available and are listed in the highest category under which they qualify (IUCN 2017b). Accordingly, we assessed all species based on the 6 criteria that can be informed by land cover and land‐cover change and classified them in the most threatened category in which they qualified. These included criteria A2 (population reduction in the last 10 years or 3 generations, whichever is longer) and B1 (small extent of occurrence [EOO]) and B2 (area of occupancy [AOO]) in combination with subcriteria a (severe fragmentation) and biii (continuing decline in habitat area, habitat extent, and/or quality of habitat), C1 (small population size and decline), D1 (very small population size), and D2 (very small AOO and plausible threats). Altogether, these criteria are currently used for the classification of 68% of threatened birds and 84% of threatened mammals. These criteria permit the classification of species into 3 threatened categories: vulnerable (VU), endangered (EN), and critically endangered (CR). If the species does not qualify under any of these categories, the species is classified as LC or near threatened (NT). However, we did not consider the NT category because it lacks explicit quantitative criteria akin to those for the threatened categories and has thus been applied less consistently across different taxa.

### Input Data

We considered all nonpelagic birds and terrestrial mammals with data on habitat preferences: total of 10,378 bird and 4,835 mammal species. We excluded pelagic birds (*n* = 362) because they spend most of the time in the open ocean far from land, and only return to very specific locations on land to breed, typically on rocky islands or coastal cliffs, where land‐cover change is generally not the major threat and for which analysis of land‐cover change is therefore not particularly informative and is extremely sensitive to the resolution used. We used the area of a minimum convex polygon encompassing the distribution maps from the IUCN Red List (IUCN 2017a; BirdLife International 2017a) to estimate the EOO (Joppa et al. 2016; IUCN 2017b). For migratory birds where polygons are classified as “resident,” “breeding range,” and “nonbreeding range,” the EOO was equal to the smallest between 2 minimum convex polygons encompassing the resident and breeding range or the resident and nonbreeding range (IUCN 2017b). We clipped the EOO maps by suitable habitat for each species based on habitat preferences coded against the IUCN habitats classification scheme (IUCN 2018). We used habitats listed in level 2 of the scheme that were coded as “suitable” for each species and excluded land at unsuitable altitudes within species ranges with data on altitudinal preferences from the IUCN Red List and the EarthEnv‐DEM90 digital elevation model (Robinson et al. 2014). To reclassify the suitable habitat and calculate the “extent of suitable habitat” (or ESH in previous studies), we used land‐cover data downloaded from https://www.esa-landcover-cci.org/ (ESA 2017) and matched it to the IUCN habitat classification scheme in level 2 following the crosswalk (i.e., conversion table) in Supporting Information. There were 536 bird and 74 mammal species for which altitudinal or habitat preferences did not result in any suitable habitat within the range, indicating possible errors in either the habitat preferences classes, altitudinal tolerances, or the match between IUCN and ESA CCI categories. This was particularly a problematic for insular bird species. Therefore, we excluded these species, resulting in a final data set of 9,842 birds and 4,761 mammals.

Each species' ESH was used in 2 ways relevant to the application of IUCN Red List criteria (Supporting Information). First, an upper estimate of the potential AOO was estimated by resampling the ESH from 300‐m to 2‐km resolution so that any 2‐km cell intersecting at least 1,300‐m cell contributed to the AOO. This follows the standardized procedure to harmonize assessments across taxa with distributional data mapped at different resolutions (IUCN 2017b). Second, we estimated the potential population size within the ESH with population‐density models based on trait information (body mass and diet), local environmental conditions (primary productivity and climatic conditions), and taxonomic information at the level of the models’ random effects (detailed explanation of the procedure in Supporting Information) (Santini et al. 2018). Although the AOO and the suitable habitat used to estimate abundance were derived from the same habitat suitability maps (ESH), it was possible that their trend was inconsistent, for instance when AOO is insensitive to changes in the amount of habitat within each 2 × 2 km grid cell.

### Red List Assessment

Under criterion A2, population changes should be calculated over 3 generations or 10 years, whichever is longer. Currently, ESA land‐cover maps are available for 1992–2015. We took 2015 as the present and identified the start point for estimating trends as 3 generations or 10 years prior to 2015, whichever was earlier. If the 3‐generation period was longer than 24 years, we calculated the change from 1992 and normalized the change as

(1) % change =% change 2015−1992·3 generations 24 years .

Generation length data for all bird and mammal species were obtained from BirdLife International (2017b) and Pacifici et al. (2013), respectively.

**Figure 1:**
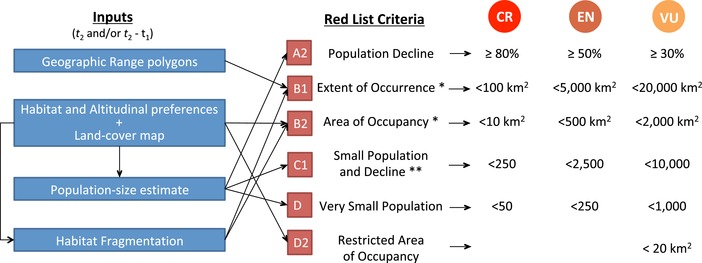
Schematic framework for prediction of species’ International Union for Conservation of Nature Red List category. All IUCN Red List criteria are applied, but only the one(s) indicating the most threatened category are used. Species that do not qualify as critically endangered (CR), endangered (EN), or vulnerable (VU) are classified as nonthreatened (*t*_1_, 2015—the longest between 3 generations and 10 years; *t*_2_, 2015; additional requirements: ^*^, subcriterion a [severely fragmented range]; subcriterion b [continuing decline in area, extent, and/or quality of habitat] must be met; ^*^^*^, decline is measured as continuing population decline ≥25% over 3 years or 1 generation for CR, ≥20% over 5 years or 2 generations for EN, and ≥10% over 10 years or 3 generations for VU).

Criteria B1 and B2 were applied by comparing the EOO and ESH estimates with the EOO and AOO thresholds, respectively (Fig. 1 & Supporting Information). However, the application of these 2 criteria requires at least 2 subcriteria to be met. We considered subcriterion a (severe fragmentation) and biii (continuing decline in area, extent, and quality of habitat or quality of habitat). *Severe fragmentation* is defined by the IUCN Red List as occurring when “increased extinction risks to the taxon results from the fact that most of its individuals are found in small and relatively isolated subpopulations” (IUCN 2017b). Accordingly, we considered species’ habitat to be fragmented if >50% of the ESH occurred in small and isolated patches (IUCN 2017b). *Small* is not defined, and varies between species according to their typical population density and other characteristics. We therefore tested multiple criteria, with *small* defined as fragments <100 km^2^; fragments supporting <100, <500, <1000, or <5000 individuals according to our population density models (Santini et al. 2018); or fragments supporting less than a viable population size according to Hilbers et al. (2016) based on viability targets with 5 assumed proportions of the maximum population growth rate (0.2, 0.4, 0.6, 0.8, or 1). Population viability estimates are only available for mammals (Hilbers et al. 2016), so in total we tested 5 definitions for birds and 10 for mammals (Supporting Information). We defined *isolated* following the approach described in Santini et al. (2014). Briefly, the approach clusters habitat in contiguous habitat fragments and then clumps those fragments within a median dispersal distance (dispersal distance estimation in Supporting Information). The resulting clumps of habitat fragments are assumed to support demographically semi‐isolated populations. Criteria C1 and D were applied by comparing population size estimates and population trends (C1 only) with their respective thresholds (Supporting Information).

Because empirical population density and dispersal distance estimates in bats are lacking and their density can be highly clumped in space due to the location of roosting sites, we did not predict density and dispersal in bats and classified them only under criteria B1 and B2. Because of the intrinsic uncertainty in the application of this criterion, we present the results considering fragmentation separately from the main results.

Overall, we based our method on conservative (optimistic) assumptions. First, we assumed the suitable habitat (ESH) to be entirely occupied (AOO), knowing that this is unrealistic. Second, our predicted abundance estimates were applied to the entire ESH and, as specified under criteria C and D, and are assumed to only represent mature individuals, therefore likely overestimating the number of these. Third, we assumed that fragmentation results only from the size of habitat fragments and their degree of isolation. Therefore our predictions are expected to be more optimistic than published IUCN Red List assessments based on empirical species‐specific data on average.

We compared predicted IUCN Red List categories with published categories by testing the correlation between ordinal values with Goodman and Kruskal's gamma statistics. We also compared the ability to detect threatened and nonthreatened species with sensitivity, specificity, and true skill statistics (TSS) (Supporting Information).

We conducted all GIS analyses with a Mollweide equal‐area projection in GRASS GIS version 7.4 (GRASS Development Team 2017) and all further statistical analyses and data processing in R version 3.5.1 (R Core Team 2018). The GRASS and R codes are available from the corresponding author upon request.

## Results

### IUCN Red List Categories Predictions

**Figure 2:**
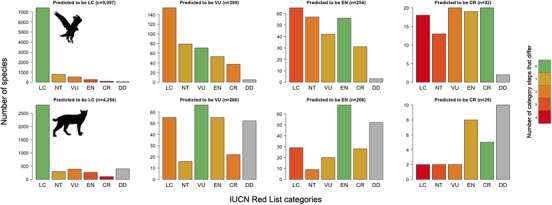
Consistency between predicted and published IUCN Red List categories for birds and mammals (LC, least concern; NT, near threatened; VU, vulnerable; EN, endangered; CR, critically endangered). Predictions make no distinction between NT and LC because no quantitative explicit criterion threshold exists for NT.

**Figure 3:**
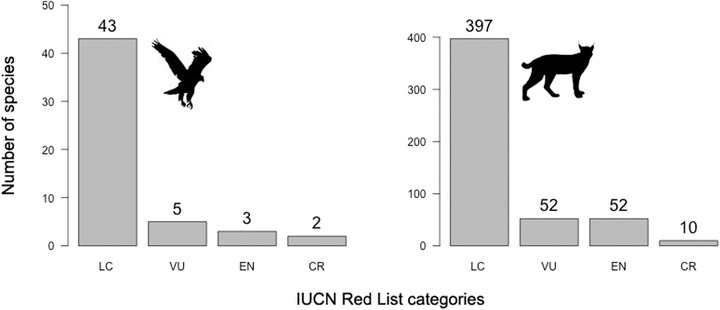
Predicted IUCN Red List categories for data‐deficient species (LC, least concern; NT, near threatened; VU, vulnerable; EN, endangered; CR, critically endangered).

We predicted 745 bird (399 VU, 254 EN, and 92 CR) and 501 mammal (266 VU, 206 EN, and 29 CR) species to be threatened (Fig. 2). These species qualified as threatened primarily under criterion B1 (53.3%, 393 birds and 348 mammals), B2 (23.4%, 165 birds and 161 mammals), D/D1 (15%, 208 birds and 70 mammals), C1 (5.6%, 59 birds and 19 mammals), and A2 (2.7%, 25 bird and 12 mammals). Among data deficient species, we predicted 10 species of birds (18.9% of bird data deficient species) and 114 of mammal species (22.3%) to be threatened (birds: 5 VU, 3 EN, and 2 CR; mammals: 52 VU, 52 EN, and 10 CR) (Fig. 3). Predictions for data deficient were concordant with those produced by previous authors using alternative methods for 76.2% of birds and 56.6% of mammals (Supporting Information). Predictions for all species are in Supporting Information.

Applying subcriterion B1a or B2a (severe fragmentation) substantially reduced the number of species qualifying under criteria B1 and B2 and hence the number of species qualifying as threatened (3.3–5.1%). The extent of this reduction depended on the quantitative definition of fragmentation applied, but overall as the minimum population size per fragment increased, the number of species qualifying as threatened under criteria B1 and B2 decreased (Supporting Information).

### Comparison Between Predicted and Published IUCN Red List Categories

Our predictions tended to be more optimistic but fairly consistent with the IUCN Red List assessments (Fig. 2). The correlation with the published IUCN Red List categories was high; birds had a G‐K gamma = 0.75 (*p* < 0.001) and mammals had a G‐K gamma = 0.74 (*p* < 0.001). The sensitivity in predicting threatened categories was low both in birds and mammals (0.29 and 0.27), and the specificity was high (0.95 and 0.96); TSS was 0.24 for birds and 0.23 for mammals. These values indicated a high type II error and low type I error (i.e., high chance of classifying a threatened species as nonthreatened, but low chance of classifying a nonthreatened species as threatened).

**Figure 4:**
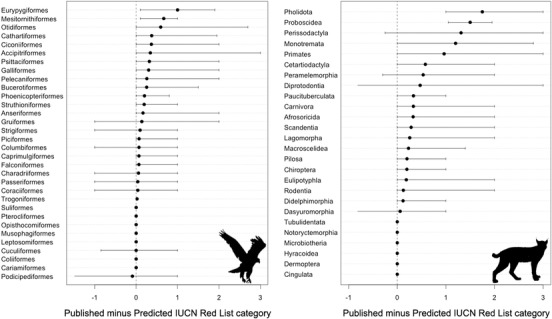
Mean difference between published and predicted IUCN Red List categories by taxonomic order for birds and mammals (least concern and near threatened = 0; vulnerable = 1; endangered = 2; critically endangered = 3). Positive values indicate predicted IUCN Red List categories are lower than current published categories on the IUCN Red List. Error bars encompass 90% of the distribution of the differences.

For birds, 467 species (4.7%) were predicted to be in higher (i.e., more threatened) IUCN Red List categories and 990 species (10%) in lower IUCN Red List categories. For mammals, 143 (3%) were predicted in higher IUCN Red List categories and 862 (18.1%) in lower IUCN Red List categories. Birds predicted to be more threatened than on the IUCN Red List qualified as threatened under criteria B1 (*n* = 276), D (*n* = 125), B2 (*n* = 99), C1 (*n* = 46), and A2 (*n* = 17). Mammals predicted to be in higher IUCN Red List categories qualified as threatened under B1 (*n* = 86), B2 (*n* = 52), D (*n* = 17), C1 (*n* = 7), and A2 (*n* = 2). The mismatches between our predictions and published IUCN Red List assessments were taxonomically biased, especially for mammals (Fig. 4). Bird species that were consistently predicted to be less threatened than on the IUCN Red List were ground‐dwelling species (e.g., Eurypygiformes, Mesitornithiformes, and Galliformes), large‐bodied species (e.g., Bucerotiformes, Ciconiiformes, and Otidiformes), and birds of prey (e.g., Accipitriformes), which are threatened by hunting, poisoning, and collision with power lines and wind turbines. Mammal species that were consistently predicted to be less threatened than currently assessed on the IUCN Red List were mostly large‐bodied species and species threatened by hunting and illegal trade (e.g., Proboscidea, Perissodactyla, Cetartiodactyla, Pholidota, and Primates) (Fig. 4).

**Figure 5:**
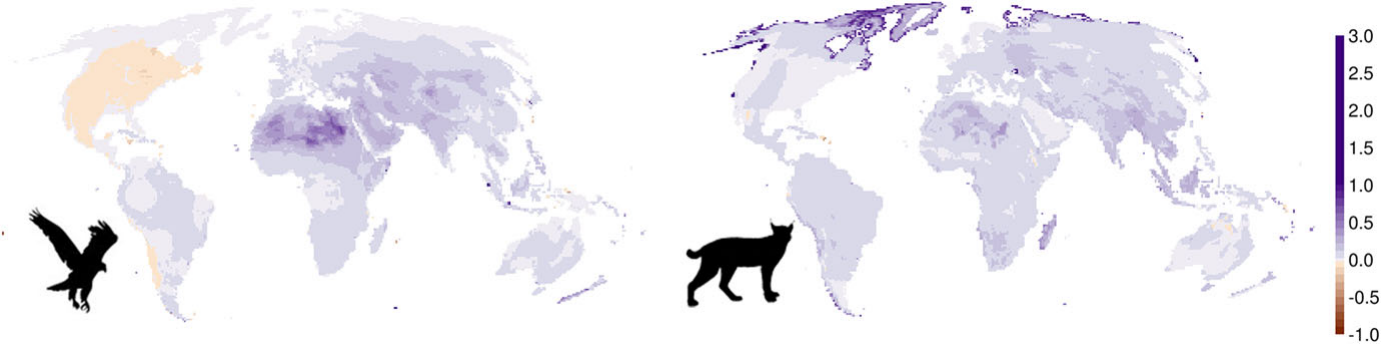
Mean difference between published and predicted IUCN Red List categories for birds and mammals per grid cell at 0.5° resolution (least concern and near threatened = 0; vulnerable = 1; endangered = 2; critically endangered = 3; positive values, predicted categories are on average lower than published categories; gray, cells with no difference on average, i.e., 0 values).

Among geographic regions, our models predicted lower extinction risk on average in the Saharo–Arabian and Australian region for birds, in the Madagascan and the Oceanian regions for mammals, and in the Oriental region for both birds and mammals. For mammals, the northern part of Alaska and Greenland also showed high values, but these areas are occupied by only a small number of species (Fig. 5 & Supporting Information). The difference between IUCN Red List assessments and our predicted categories was positively correlated with species’ body mass in birds and mammals (Supporting Information), indicating that our approach is more likely to underestimate the IUCN Red List categories of larger species compared with those of smaller species. Finally, the comparison between our predictions and published assessments depended on the assumptions made for the AOO and population size (Supporting Information). A sensitivity analysis of the effect of these 2 parameters on the predictions suggested that the number of threatened species may be much higher than currently predicted (Supporting Information).

## Discussion

Our results demonstrate how data on land‐cover change coupled with information on species habitat preferences and modeled abundance can inform species assessments under the IUCN Red List. This procedure was particularly useful in detecting species whose rate and degree of habitat loss may have been underestimated. Further, it identified data‐deficient species most likely to be threatened, thus targeting research designed to gather sufficient information to apply the IUCN Red List criteria. The same procedure can be applied to other taxonomic groups for which distributions have been mapped, habitat preferences documented, and abundance predictions can be made (e.g., Amphibians; Ficetola et al. 2015; Santini et al. 2018). Our predictions were fairly consistent with published IUCN Red List assessments, suggesting that the procedure is reliable for preliminary species assessments. However, our approach also classified many species in higher or lower IUCN Red List categories than their published status, highlighting limitations and strengths in both the IUCN Red List and our predictive method.

Most mismatches between published and predicted status involved species predicted to be less threatened than they are classified on the IUCN Red List. These mismatches include many species for which the assumption of presence in suitable habitat is particularly overoptimistic because several factors other than habitat type can determine their presence (Colwell & Rangel 2009). For example, some species show clumped distributions within suitable habitat due to the patchy distribution of key resources (Mayor et al. 2009) or else shift nomadically around their geographic range and thus occupy only a limited part of it at any given time (Runge et al. 2015). Similarly, some species are absent or rarer in areas subject to particular threats, such as overexploitation, invasive alien species, pollution, or human disturbance (Benítez‐López et al. 2010, 2017; Hoffmann et al. 2010; Ripple et al. 2016). Pangolins (Pholidota), for instance, are the most heavily trafficked mammals in the world and are facing severe population declines due to overexploitation in Asia and Africa. Pangolins can occur in a large variety of habitats, including primary and secondary tropical forests, shrublands, and grasslands; therefore, their ESH is large, yet their AOO is likely to be considerably smaller (Ingram et al. 2017). Our predictions for small species were more consistent with the IUCN Red List than those for large species, probably because the latter are more often threatened by direct exploitation, whereas smaller species are more often threatened by habitat loss and degradation (Ripple et al. 2017).

Our analysis revealed a consistent bias toward underestimation of risk in the predictions and overestimation in the assessments for the Madagascan, Oriental, and Oceanian regions in mammals, and in the Oriental, Saharo–Arabian, and Australian regions in birds (Fig. 5 & Supporting Information). These mismatches can be explained by a combination of factors including high hunting pressure (e.g., Oriental), threats from invasive species (e.g., Australian and Oceanian), or climate change (e.g., Saharo–Arabian) in these regions (Loarie et al. 2009; Benítez‐López et al. 2017; Spatz et al. 2017). Mismatches between predictions and IUCN Red List assessments appear to be larger and more taxonomically and geographically biased in mammals (Fig. 4 & Supporting Information). This may arise from underlying methods because bird assessments are all performed by BirdLife International, whereas mammal assessments are coordinated by the Global Mammal Assessment but in effect generated by the many specialist groups for different taxa worldwide. This can result in inconsistencies between different taxonomic groups, for example, in the application of different criteria, use of different types of data sources, or in evidentiary versus precautionary attitudes.

We consider our approach particularly useful for species and regions that receive less research attention (Donaldson et al. 2016; Verde Arregoitia 2016; Di Marco et al. 2017) because they are often assessed by a small number of experts based on a limited amount of data collated from old publications or anecdotal information. For example, the Polar Bear Specialist Group has 25 members (http://pbsg.npolar.no/en/), whereas the Small Mammals Specialist Group, which overall covers around >2800 species, has around 120 members (http://www.small-mammals.org/). This inevitably influences the quality of the assessments. Overall, most species on the IUCN Red List are not assessed against all criteria, owing to lack of data (IUCN 2017b).

Although considering only 1 or a few criteria allows poorly known species to be assessed, it also makes the IUCN Red List sensitive to data availability. In fact, species assessed against more criteria are more likely to be classified as threatened. Because we simultaneously assessed species under 5 different criteria that are based on up‐to‐date remote‐sensing information, it is possible that our approach identifies species that are genuinely threatened at present, but have not yet been assessed as such on the IUCN Red List. For example, many species that could be classified as threatened based on B1 (i.e., EOO smaller than the required thresholds) are not classified as such on the IUCN Red List because they are considered to have stable population trends (subcriterion biii). However, we classified some of these as threatened under B1 because land‐cover data indicated that they have lost habitat over the last 10 years or 3 generations. Two examples are the Northern Ground‐hornbill (*Bucorvus abyssinicus*) and the Ethiopian striped mouse (*Muriculus imberbis*), which are poorly known and have restricted geographical ranges but are believed to be stable in terms of distribution and population size. Both species are currently classified as LC, but our models predicted that they have undergone a severe decline in ESH and population size and should therefore qualify as EN (Supporting Information). Review of the IUCN Red List assessments for species such as these are now warranted and should involve targeted efforts to compile up‐to‐date information on their status.

A useful application of our approach is the preliminary assessment of data deficient species. Although there are few data deficient birds (0.6% of species), around 14% of mammal species are classified as data deficient. Our approach identified several species in urgent need of conservation attention, such as the Brown‐banded Rail (*Lewinia mirifica*) and Williamson's mouse‐deer (*Tragulus williamsoni*), which were predicted to be CR by our models. Our predictions for data deficient species were fairly consistent with those based on expert judgment and machine‐learning algorithms (Supporting Information; Butchart & Bird 2010; Bland et al. 2015). Our approach mostly relies on changes in habitat availability over time, while Bland et al. (2015) primarily focused on species’ intrinsic vulnerability to extinction; therefore these 2 approaches offer different, and complementary, perspectives on identifying species that require urgent monitoring and targeted research.

The number of species qualifying as threatened in our analyses was substantially reduced when the fragmentation subcriterion was applied, depending on the threshold used to define small fragments. This raises the issue that the IUCN definition of habitat fragmentation is qualitative and can be interpreted in different ways (IUCN 2017b). As a consequence, the fragmentation subcriterion is typically applied based on expert opinion, which may imply that many species currently listed under criterion B should not be considered as threatened. Our operative interpretation of the definition allows this criterion to be applied more objectively and consistently across species (Santini et al. 2014; Di Marco et al. 2016), but it is also sensitive to the land‐cover data resolution, which may be inappropriate for many species and ignores the effect of barriers. This may result in excessively conservative assessments, perhaps explaining the reduced number of a species qualifying under this subcriterion. We therefore urge IUCN to provide more explicit guidance on how to apply this subcriterion more objectively and consistently, including through quantitative approaches such as ours.

Our results show how the Red List framework can be applied using land‐cover maps coupled with information on habitat preferences and spatially explicit abundance models. Because this approach tends to underestimate species extinction risk, it implies that any species predicted to be more at risk than currently classified should be urgently reassessed. We propose that this approach be integrated into IUCN Red List assessments to reduce taxonomic and spatial biases and to address constraints in data availability. More importantly, as periodic updates of automatically processed satellite images become available, our approach can be automated to provide an early‐warning system to identify species potentially warranting urgent conservation actions.

## Supporting information

Supplementary Methods (Appendix S1) and Supplementary Results (Appendix S2) are available online. The authors are solely responsible for the content and functionality of these materials. Queries (other than absence of the material) should be directed to the corresponding author.
