# Supplementary material for: Applying habitat and population‐density models to land‐cover time series to inform IUCN Red List assessments
Source: Conserv Biol. 2019 Feb 25;33(5):1084–93. doi: 10.1111/cobi.13279 (PMC6767507; doi:10.1111/cobi.13279)
Supplement: Supplementary file 1 — Supplementary Methods (Appendix S1) and Supplementary Results (Appendix S2) are available online. The authors are solely responsible for the content and functionality of these materials. Queries (other than absence of the material) should be directed to the corresponding author. [file COBI-33-1084-s001.pdf]

Supporting Information

Applying habitat and population-density models to land-cover time series to inform IUCN Red List

Luca Santini [1]*, Stuart H. M. Butchart [2,3], Carlo Rondinini [4], Ana Benítez-López [1], Jelle P. Hilbers [5], Aafke M. Schipper [1,5], Mirza Cengic [1], Joseph A. Tobias [6], Mark A. J. Huijbregts [1]

[1] Department of Environmental Science, Institute for Wetland and Water Research, Faculty of Science, Radboud University, P.O. Box
9010, NL-6500 GL, Nijmegen, The Netherlands

[2] BirdLife international, David Attenborough Building, Pembroke Street, Cambridge, CB23QZ, U.K.

[3] Department of Zoology, University of Cambridge, Downing Street, Cambridge, CB23EJ, U.K.

[4] Department of Biology and Biotechnologies, Sapienza Universit`a di Roma, Viale dell'Universit`a 32, 00185, Rome, Italy

[5] PBL Netherlands Environmental Assessment Agency, PO Box 30314, 2500 GH, The Hague, The Netherlands

[6] Department of Life Sciences, Imperial College London, Silwood Park, Buckhurst Road, Ascot, Berkshire, SL5 7PY, U.K.

* email luca.santini.eco@gmail.com

Appendix S1. Supplementary methods

Table S1. Quantitative thresholds for each IUCN Red List category and the Red List criteria applied in this study (source IUCN 2017).

Criterion	Focus	Critically Endangered	Endangered	Vulnerable	Additional requirements
**A2**	Population Decline	≥ 80%	≥ 50%	≥ 30%	
**B1**	Extent of Occurrence	<100 km^2	<5,000 km^2	<20,000 km^2	Subcriteria a (severely fragmented range) and b (continuing decline in area/extent and/or quality of habitat)
**B2**	Area of Occupancy	<10 km^2	<500 km^2	<2000 km^2	
**C1**	Small Population and Decline	<250 mature individuals	<2,500 mature individuals	<10,000 mature individuals	Continuing population decline ≥25% over 3 yrs/1 generation for Critically Endangered, ≥20% over 5yrs/2 generations for Endangered, ≥10% over 10 yrs/3 generations for Vulnerable).
**D**	Very Small Populations	<50 mature individuals	<250 mature individuals	<1,000 mature individuals	
**D2**	Restricted AOO			typically: AOO <20 km2 or number of locations ≤ 5	Restricted area of occupancy or number of locations with a plausible future threat that could drive the taxon to CR or EX in a very short time.

Table S2. Cross-walk between IUCN Habitat classification scheme at level 2 and ESA CCI land cover categories.

IUCN - code	IUCN - Habitat description	ESA CCI – code	ESA CCI – Habitat description
1	Forest - Boreal	60	Tree cover, broadleaved, deciduous, closed to open (>15%)
1.1	Forest - Boreal	61	Tree cover, broadleaved, deciduous, closed (>40%)
1.1	Forest - Boreal	62	Tree cover, broadleaved, deciduous, open (15-40%)
1.1	Forest - Boreal	70	Tree cover, needleleaved, evergreen, closed to open (>15%)
1.1	Forest - Boreal	71	Tree cover, needleleaved, evergreen, closed (>40%)
1.1	Forest - Boreal	72	Tree cover, needleleaved, evergreen, open (15-40%)
1.1	Forest - Boreal	80	Tree cover, needleleaved, deciduous, closed to open (>15%)
1.1	Forest - Boreal	81	Tree cover, needleleaved, deciduous, closed (>40%)
1.1	Forest - Boreal	82	Tree cover, needleleaved, deciduous, open (15-40%)
1.1	Forest - Boreal	90	Tree cover, mixed leaf type (broadleaved and needleleaved)
1.2	Forest - Subarctic	60	Tree cover, broadleaved, deciduous, closed to open (>15%)
1.2	Forest - Subarctic	62	Tree cover, broadleaved, deciduous, open (15-40%)
1.2	Forest - Subarctic	70	Tree cover, needleleaved, evergreen, closed to open (>15%)
1.2	Forest - Subarctic	72	Tree cover, needleleaved, evergreen, open (15-40%)
1.2	Forest - Subarctic	80	Tree cover, needleleaved, deciduous, closed to open (>15%)
1.2	Forest - Subarctic	82	Tree cover, needleleaved, deciduous, open (15-40%)
1.3	Forest - Subantarctic	60	Tree cover, broadleaved, deciduous, closed to open (>15%)
1.3	Forest - Subantarctic	62	Tree cover, broadleaved, deciduous, open (15-40%)
1.3	Forest - Subantarctic	70	Tree cover, needleleaved, evergreen, closed to open (>15%)
1.3	Forest - Subantarctic	72	Tree cover, needleleaved, evergreen, open (15-40%)

IUCN - code	IUCN - Habitat description	ESA CCI – code	ESA CCI – Habitat description
1.3	Forest - Subantarctic	80	Tree cover, needleleaved, deciduous, closed to open (>15%)
1.3	Forest - Subantarctic	82	Tree cover, needleleaved, deciduous, open (15-40%)
1.4	Forest - Temperate	50	Tree cover, broadleaved, evergreen, closed to open (>15%)
1.4	Forest - Temperate	60	Tree cover, broadleaved, deciduous, closed to open (>15%)
1.4	Forest - Temperate	61	Tree cover, broadleaved, deciduous, closed (>40%)
1.4	Forest - Temperate	62	Tree cover, broadleaved, deciduous, open (15-40%)
1.4	Forest - Temperate	70	Tree cover, needleleaved, evergreen, closed to open (>15%)
1.4	Forest - Temperate	71	Tree cover, needleleaved, evergreen, closed (>40%)
1.4	Forest - Temperate	72	Tree cover, needleleaved, evergreen, open (15-40%)
1.4	Forest - Temperate	80	Tree cover, needleleaved, deciduous, closed to open (>15%)
1.4	Forest - Temperate	81	Tree cover, needleleaved, deciduous, closed (>40%)
1.4	Forest - Temperate	82	Tree cover, needleleaved, deciduous, open (15-40%)
1.4	Forest - Temperate	90	Tree cover, mixed leaf type (broadleaved and needleleaved)
1.5	Forest - Subtropical/Tropical Dry	60	Tree cover, broadleaved, deciduous, closed to open (>15%)
1.5	Forest - Subtropical/Tropical Dry	62	Tree cover, broadleaved, deciduous, open (15-40%)
1.5	Forest - Subtropical/Tropical Dry	70	Tree cover, needleleaved, evergreen, closed to open (>15%)
1.5	Forest - Subtropical/Tropical Dry	72	Tree cover, needleleaved, evergreen, open (15-40%)
1.6	Forest - Subtropical/Tropical Moist Lowland	50	Tree cover, broadleaved, evergreen, closed to open (>15%)
1.6	Forest - Subtropical/Tropical Moist Lowland	60	Tree cover, broadleaved, deciduous, closed to open (>15%)
1.6	Forest - Subtropical/Tropical Moist Lowland	61	Tree cover, broadleaved, deciduous, closed (>40%)
1.7	Forest - Subtropical/Tropical Mangrove Vegetation Above High Tide Level	170	Tree cover, flooded, saline water

IUCN - code	IUCN - Habitat description	ESA CCI – code	ESA CCI – Habitat description
1.7	Forest - Subtropical/Tropical Mangrove Vegetation Above High Tide Level	180	Shrub or herbaceous cover, flooded, fresh/saline/brakish water
1.8	Forest - Subtropical/Tropical Swamp	160	Tree cover, flooded, fresh or brakish water
1.9	Forest - Subtropical/Tropical Moist Montane	50	Tree cover, broadleaved, evergreen, closed to open (>15%)
1.9	Forest - Subtropical/Tropical Moist Montane	71	Tree cover, needleleaved, evergreen, closed (>40%)
1.9	Forest - Subtropical/Tropical Moist Montane	90	Tree cover, mixed leaf type (broadleaved and needleleaved)
2.1	Savanna - Dry	110	Mosaic herbaceous cover (>50%) / tree and shrub (<50%)
2.1	Savanna - Dry	120	Shrubland
2.1	Savanna - Dry	122	Deciduous shrubland
2.1	Savanna - Dry	130	Grassland
2.2	Savanna - Moist	180	Shrub or herbaceous cover, flooded, fresh/saline/brakish water
2.2	Savanna - Moist	100	Mosaic tree and shrub (>50%) / herbaceous cover (<50%)
2.2	Savanna - Moist	110	Mosaic herbaceous cover (>50%) / tree and shrub (<50%)
3.1	Shrubland - Subarctic	120	Shrubland
3.1	Shrubland - Subarctic	121	Evergreen shrubland
3.1	Shrubland - Subarctic	122	Deciduous shrubland
3.2	Shrubland - Subantarctic	120	Shrubland
3.2	Shrubland - Subantarctic	121	Evergreen shrubland
3.2	Shrubland - Subantarctic	122	Deciduous shrubland
3.3	Shrubland - Boreal	120	Shrubland
3.3	Shrubland - Boreal	121	Evergreen shrubland
3.3	Shrubland - Boreal	122	Deciduous shrubland

IUCN - code	IUCN - Habitat description	ESA CCI – code	ESA CCI – Habitat description
3.4	Shrubland - Temperate	120	Shrubland
3.4	Shrubland - Temperate	121	Evergreen shrubland
3.4	Shrubland - Temperate	122	Deciduous shrubland
3.5	Shrubland - Subtropical/Tropical Dry	120	Shrubland
3.5	Shrubland - Subtropical/Tropical Dry	121	Evergreen shrubland
3.5	Shrubland - Subtropical/Tropical Dry	122	Deciduous shrubland
3.6	Shrubland - Subtropical/Tropical Moist	120	Shrubland
3.6	Shrubland - Subtropical/Tropical Moist	121	Evergreen shrubland
3.7	Shrubland - Subtropical/Tropical High Altitude	120	Shrubland
3.7	Shrubland - Subtropical/Tropical High Altitude	121	Evergreen shrubland
3.7	Shrubland - Subtropical/Tropical High Altitude	122	Deciduous shrubland
3.8	Shrubland - Mediterranean-type Shrubby Vegetation	120	Shrubland
3.8	Shrubland - Mediterranean-type Shrubby Vegetation	121	Evergreen shrubland
3.8	Shrubland - Mediterranean-type Shrubby Vegetation	122	Deciduous shrubland
4.1	Grassland - Tundra	130	Grassland
4.1	Grassland - Tundra	140	Lichens and mosses
4.1	Grassland - Tundra	150	Sparse vegetation (tree, shrub, herbaceous cover) (<15%)
4.1	Grassland - Tundra	152	Sparse shrub (<15%)
4.1	Grassland - Tundra	180	Shrub or herbaceous cover, flooded, fresh/saline/brakish water
4.2	Grassland - Subarctic	130	Grassland
4.2	Grassland - Subarctic	140	Lichens and mosses
4.2	Grassland - Subarctic	150	Sparse vegetation (tree, shrub, herbaceous cover) (<15%)

IUCN - code	IUCN - Habitat description	ESA CCI – code	ESA CCI – Habitat description
4.2	Grassland - Subarctic	180	Shrub or herbaceous cover, flooded, fresh/saline/brakish water
4.3	Grassland - Subantarctic	130	Grassland
4.4	Grassland - Temperate	130	Grassland
4.5	Grassland - Subtropical/Tropical Dry	130	Grassland
4.6	Grassland - Subtropical/Tropical Seasonally Wet/Flooded	180	Shrub or herbaceous cover, flooded, fresh/saline/brakish water
4.7	Grassland - Subtropical/Tropical High Altitude	130	Grassland
5.1	Wetlands (inland) - Permanent Rivers/Streams/Creeks (includes waterfalls)	210	Water bodies
5.1	Wetlands (inland) - Tundra Wetlands (incl. pools and temporary waters from snowmelt)	210	Water bodies
5.11	Wetlands (inland) - Alpine Wetlands (includes temporary waters from snowmelt)	210	Water bodies
5.12	Wetlands (inland) - Geothermal Wetlands	210	Water bodies
5.13	Wetlands (inland) - Permanent Inland Deltas	210	Water bodies
5.14	Wetlands (inland) - Permanent Saline, Brackish or Alkaline Lakes	210	Water bodies
5.15	Wetlands (inland) - Seasonal/Intermittent Saline, Brackish or Alkaline Lakes and Flats	210	Water bodies
5.16	Wetlands (inland) - Permanent Saline, Brackish or Alkaline Marshes/Pools	210	Water bodies
5.17	Wetlands (inland) - Seasonal/Intermittent Saline, Brackish or Alkaline Marshes/Pools	210	Water bodies
5.18	Wetlands (inland) - Karst and Other Subterranean Hydrological Systems (inland)	NA	NA
5.2	Wetlands (inland) - Seasonal/Intermittent/Irregular Rivers/Streams/Creeks	210	Water bodies
5.3	Wetlands (inland) - Shrub Dominated Wetlands	180	Shrub or herbaceous cover, flooded, fresh/saline/brakish water
5.4	Wetlands (inland) - Bogs, Marshes, Swamps, Fens, Peatlands	180	Shrub or herbaceous cover, flooded, fresh/saline/brakish water

IUCN - code	IUCN - Habitat description	ESA CCI – code	ESA CCI – Habitat description
5.5	Wetlands (inland) - Permanent Freshwater Lakes (over 8ha)	210	Water bodies
5.6	Wetlands (inland) - Seasonal/Intermittent Freshwater Lakes (over 8ha)	210	Water bodies
5.7	Wetlands (inland) - Permanent Freshwater Marshes/Pools (under 8ha)	210	Water bodies
5.8	Wetlands (inland) - Seasonal/Intermittent Freshwater Marshes/Pools (under 8ha)	210	Water bodies
5.9	Wetlands (inland) - Freshwater Springs and Oases	210	Water bodies
6	Rocky areas (eg. inland cliffs, mountain peaks)	200	Bare areas
6	Rocky areas (eg. inland cliffs, mountain peaks)	201	Consolidated bare areas
7.1	Caves and Subterranean Habitats (non-aquatic) - Caves	NA	NA
7.2	Caves and Subterranean Habitats (non-aquatic) - Other Subterranean Habitats	NA	NA
8.1	Desert - Hot	200	Bare areas
8.1	Desert - Hot	201	Consolidated bare areas
8.1	Desert - Hot	202	Unconsolidated bare areas
8.1	Desert - Hot	150	Sparse vegetation (tree, shrub, herbaceous cover) (<15%)
8.1	Desert - Hot	153	Sparse herbaceous cover (<15%)
8.2	Desert - Temperate	200	Bare areas
8.2	Desert - Temperate	201	Consolidated bare areas
8.2	Desert - Temperate	202	Unconsolidated bare areas
8.2	Desert - Temperate	150	Sparse vegetation (tree, shrub, herbaceous cover) (<15%)
8.2	Desert - Temperate	153	Sparse herbaceous cover (<15%)
8.3	Desert - Cold	200	Bare areas
8.3	Desert - Cold	201	Consolidated bare areas

IUCN - code	IUCN - Habitat description	ESA CCI – code	ESA CCI – Habitat description
8.3	Desert - Cold	202	Unconsolidated bare areas
8.3	Desert - Cold	150	Sparse vegetation (tree, shrub, herbaceous cover) (<15%)
8.3	Desert - Cold	140	Lichens and mosses
9.1	Marine Neritic - Estuaries	210	Water bodies
9.1	Marine Neritic - Pelagic	210	Water bodies
9.2	Marine Neritic - Subtidal Rock and Rocky Reefs	210	Water bodies
9.3	Marine Neritic - Subtidal Loose Rock/pebble/gravel	210	Water bodies
9.4	Marine Neritic - Subtidal Sandy	210	Water bodies
9.5	Marine Neritic - Subtidal Sandy-Mud	210	Water bodies
9.6	Marine Neritic - Subtidal Muddy	210	Water bodies
9.7	Marine Neritic - Macroalgal/Kelp	210	Water bodies
9.9	Marine Neritic - Seagrass (Submerged)	210	Water bodies
10.1	Marine Oceanic - Epipelagic (0-200m)	210	Water bodies
10.2	Marine Oceanic - Mesopelagic (200-1000m)	210	Water bodies
10.3	Marine Oceanic - Bathypelagic (1000-4000m)	210	Water bodies
12.1	Marine Intertidal - Rocky Shoreline	201	Consolidated bare areas
12.2	Marine Intertidal - Sandy Shoreline and/or Beaches, Sand Bars, Spits, Etc	202	Unconsolidated bare areas
12.3	Marine Intertidal - Shingle and/or Pebble Shoreline and/or Beaches	202	Unconsolidated bare areas
12.4	Marine Intertidal - Mud Flats and Salt Flats	202	Unconsolidated bare areas
12.5	Marine Intertidal - Salt Marshes (Emergent Grasses)	180	Shrub or herbaceous cover, flooded, fresh/saline/brakish water
12.6	Marine Intertidal - Tidepools	210	Water bodies
12.7	Marine Intertidal - Mangrove Submerged Roots	210	Water bodies

IUCN - code	IUCN - Habitat description	ESA CCI – code	ESA CCI – Habitat description
13.1	Marine Coastal/Supratidal - Sea Cliffs and Rocky Offshore Islands	201	Consolidated bare areas
13.2	Marine Coastal/supratidal - Coastal Caves/Karst	201	Consolidated bare areas
13.3	Marine Coastal/Supratidal - Coastal Sand Dunes	202	Unconsolidated bare areas
13.4	Marine Coastal/Supratidal - Coastal Brackish/Saline Lagoons/Marine Lakes	201	Consolidated bare areas
13.5	Marine Coastal/Supratidal - Coastal Freshwater Lakes	201	Consolidated bare areas
14.1	Artificial/Terrestrial - Arable Land	10	Cropland, rainfed
14.1	Artificial/Terrestrial - Arable Land	11	Cropland, rainfed, Herbaceous cover
14.1	Artificial/Terrestrial - Arable Land	12	Cropland, rainfed, Tree or shrub cover
14.1	Artificial/Terrestrial - Arable Land	20	Cropland, irrigated or post-flooding
14.2	Artificial/Terrestrial - Pastureland	130	Grassland
14.3	Artificial/Terrestrial - Plantations	12	Cropland, rainfed, Tree or shrub cover
14.4	Artificial/Terrestrial - Rural Gardens	30	Mosaic cropland (>50%) / natural vegetation (tree, shrub, herbaceous cover) (<50%)
14.4	Artificial/Terrestrial - Rural Gardens	40	Mosaic natural vegetation (tree, shrub, herbaceous cover) (>50%) / cropland (<50%)
14.5	Artificial/Terrestrial - Urban Areas	190	Urban areas
14.6	Artificial/Terrestrial - Subtropical/Tropical Heavily Degraded Former Forest	30	Mosaic cropland (>50%) / natural vegetation (tree, shrub, herbaceous cover) (<50%)
15.1	Artificial/Aquatic - Water Storage Areas (over 8ha)	210	Water bodies
15.1	Artificial/Aquatic - Karst and Other Subterranean Hydrological Systems (human-made)	NA	NA
15.2	Artificial/Aquatic - Ponds (below 8ha)	210	Water bodies
15.3	Artificial/Aquatic - Aquaculture Ponds	210	Water bodies
15.4	Artificial/Aquatic - Salt Exploitation Sites	202	Unconsolidated bare areas

IUCN - code	IUCN - Habitat description	ESA CCI – code	ESA CCI – Habitat description
15.5	Artificial/Aquatic - Excavations (open)	202	Unconsolidated bare areas
15.6	Artificial/Aquatic - Wastewater Treatment Areas	190	Urban areas
15.7	Artificial/Aquatic - Irrigated Land (includes irrigation channels)	20	Cropland, irrigated or post-flooding
15.8	Artificial/Aquatic - Seasonally Flooded Agricultural Land	20	Cropland, irrigated or post-flooding
15.9	Artificial/Aquatic - Canals and Drainage Channels, Ditches	210	Water bodies
16	Introduced vegetation	NA	NA
17	Other	NA	NA
18	Unknown	NA	NA
9.8.1	Outer Reef Channel	210	Water bodies
9.8.3	Foreslope (Outer Reef Slope)	210	Water bodies
9.8.4	Lagoon	210	Water bodies
9.8.5	Inter-Reef Soft Substrate	210	Water bodies
9.8.6	Inter-Reef Rubble Substrate	210	Water bodies

Fig. S1. Methodological steps to produce reclassify land cover (LC) maps to Extent of Suitable Habitat (ESH) within the Extent of Occurrence (EOO), in turn used to calculate the Area of Occupancy (AOO) and habitat area for the abundance estimate.

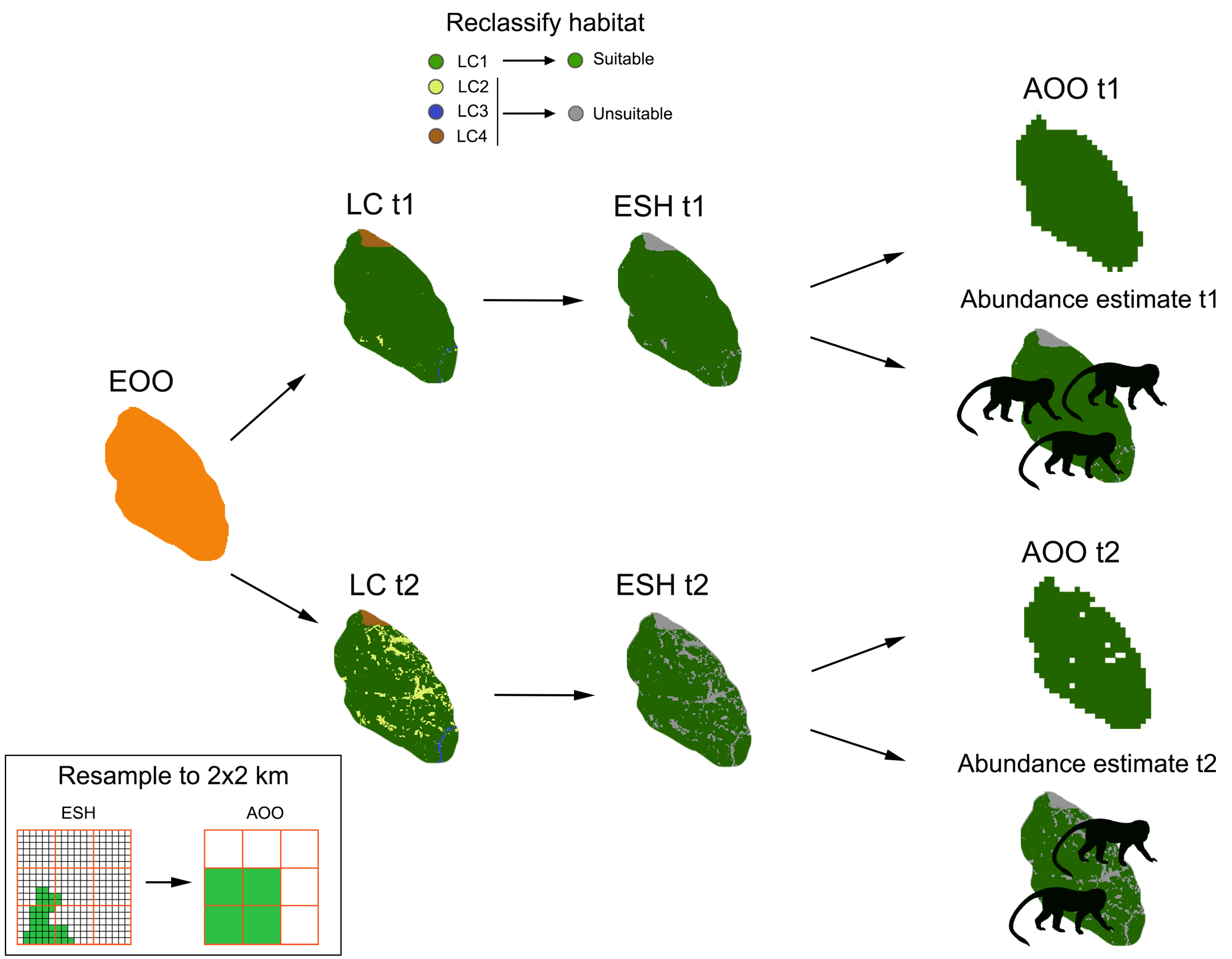

Fig. S2. Steps taken to identify the clumps of habitats fragments (Adapted from Santini et al. 2014).

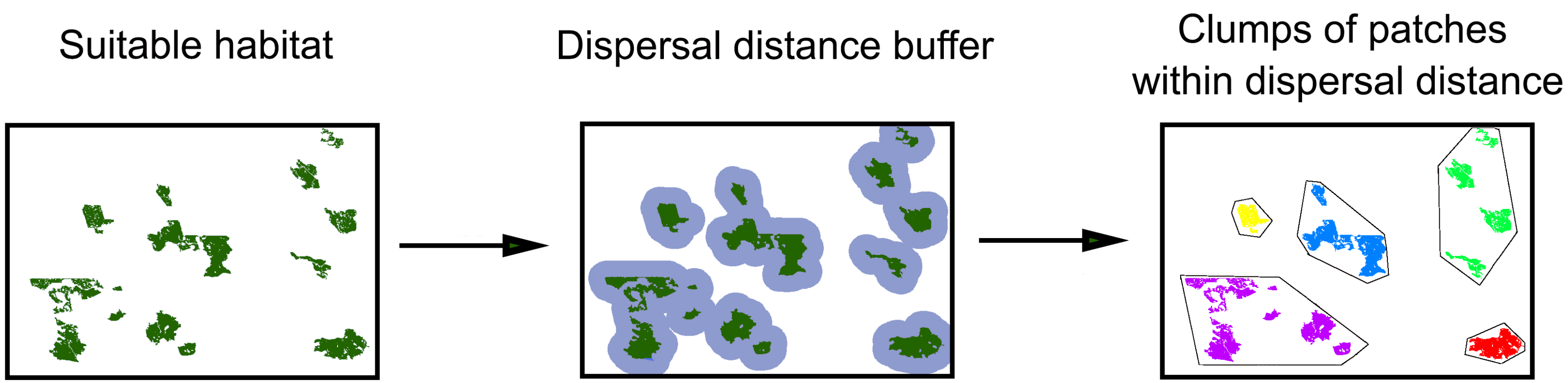

Appendix S1.1. Population abundance predictions

We predicted species population abundance within suitable habitat using population density models presented in Santini et al. (2018b). These models are mixed effect models based on trait information (body mass and diet) and local environmental conditions (primary productivity and climatic conditions), with taxonomic information included as random effects to account for phylogenetic non-independence. Among all models tested in Santini et al. (2018b), we used the best models in terms of predictive performance as measured by the minimum absolute error:

Birds:

D ~ BM + Diet + NPP + I(NPP^2) + Pwq + Pcv+ I(Pcv^2)

Mammals:

D ~ BM + Diet + NPP + I(NPP^2) + Pwq + I(Pwq^2) + Pcv + I(Pcv^2)

Where: D = log10 population density; BM = log10 body mass; Diet = Diet category (birds: Plant and seed eaters, frugivores/nectarivores, invertivores, carnivores (consuming vertebrates or carrion), omnivores; Mammals: Herbivores, Omnivores, Carnivores); NPP = log10 Net Primary Productivity; Pwq = Precipitation of the warmest quarter; Pcv = Precipitation seasonality.

As in Santini et al. (2018b), we used body mass and diet categories from Wilman et al. (2014), Net Primary Productivity from Imhoff et al. (2004) and Precipitation of the warmest quarter and precipitation seasonality from Hijman et al. (2005). In addition to the fixed effect component of the model, the predictions were also informed by the random effects that modelled deviations from the intercept for taxonomic orders, families and species hierarchically. When a taxon level for a species was not present in the models the respective random effect was set to zero.

We predicted species population density per geographic range grid cell resampled at 100 km resolution to match the resolution of the environmental variables in the fitted model (Santini et al. 2018). Because insular species tend to live at higher population densities on average, and our

predictions would largely underestimate their total population size, we adjusted the predicted densities of insular species by adding a constant to the intercept of the models. The constants were calculated as the difference between the intercepts of insular and mainland species in a model considering “insular” all species living in islands smaller 1000 km2 (including any island smaller than Greenland) (see Fig. S3 in Santini et al. 2018b). The estimated constants were equal to 0.0437919562 for birds and 0.1998995839 for mammals. For the predictions, we considered insular all species living exclusively in islands smaller than 1000 km2.

To obtain a median estimate of the population size per geographic range grid cell we multiplied the final density predictions by the total extent of suitable habitat within the grid cell. Finally, we summed the predicted population size per cell to obtain an overall estimate of the population size.

Appendix S1.2. Predictions of dispersal distance in birds and mammals

We modelled natal dispersal distance based on published dispersal estimates for 430 bird species compiled by BirdLife International. We only retained mean values from studies with $n \geq 5$, which reduced the dataset to 370 species. When estimates for both sexes were available for a species, we used the estimate for the most dispersive sex.

We considered the following predictors: Body mass (g), Wing length (mm), Hand Wing Index (HWI), Bill length (mm), Bill depth (mm), Geographic range (km^2), and Generation length (days). Body mass values were extracted from published data (Wilman et al. 2014) . Wing length, HWI, Beak length and Beak depth were all measured from museum specimens. Wing length (distance between the carpal joint to wingtip) was measured on the folded wing with primaries unflattened. Following Claramunt et al. (2012), we computed HWI as the ratio of Kipp's distance (distance between the tip of the longest primary/wingtip and the first secondary feather) to total wing length. Measurements of beak length (along the culmen to the beak tip) and depth (as vertical height) were taken at or from the anterior edge of the nostrils. In all cases, we obtained measurements from at least 4 individuals (2 from each sex) for each species, following standard protocols (Pigot & Tobias 2014; Pigot et al. 2016).

Body mass is significantly but weakly associated with animal dispersal (Sutherland et al. 2000). Similarly, the four measurements of wing and bill morphology are proxies of flight ability and diet category, and were considered in previous models of bird dispersal (Dawideit et al. 2009). HWI is a measure of wingtip shape, providing information about the narrowness and pointedness of the avian wing. HWI captures some variation in avian flight ability that is not associated with body size because species with stronger and more frequent flight tend to have narrower and more pointed wingtips. HWI is highly correlated with Kipp's distance (Stoddard et al. 2017), but scaled to wing length, thus providing a better approximation of wing aspect ratio (WAR) (Claramunt et al. 2012; Kennedy et al. 2016).

We compiled geographic range size from (BirdLife International and Handbook of the Birds of the World 2017), and generation length from BirdLife International (2017). Geographic range is related to dispersal distance in mammals (Whitmee & Orme 2012) as good dispersers tend to be widely distributed. Finally, generation length was used as a proxy of dispersal distance in mammals (Whitmee & Orme 2012). After preliminary data exploration that indicated a non-linear relationship between dispersal and Generation length and HWI, we also inclued a quadratic effect for these variables.

We assessed the collinearity between our predictors, and listed a total of 44 models, excluding formulas in which collinear variables appeared together (ρ>0.7).
We used a mixed effect model with a nested random effect structure with Order/Family/Genus.
To evaluate the predictive accuracy of the models, we ran a 5-fold cross-validation repeated 10 times for each of the model (Fig. S3), and used the results to calculate the Maximum Absolute Error (MAE). Models were compared using one information criterion, the Akaike Information Criterion, and one predictive accuracy, the Minimum Absolute Error.
The best model according to both metrics was:
Mean Dispersal Distance ~ Hand Wing Index + Body mass + Geographic Range

Modelled values of dispersal distance were used where available, with any value <0.03 km set to 0.03 km. Species with no modelled values were allocated the genus mean (832 spp), unless no value was available, in which case the family mean was used (55 spp). Two species comprising the family Turnagridae were assigned the family mean for Oriolidae to which they are most closely related. Values for Struthionidae, Rheidae, and Casuaridae were set to 10 km, and those for Apterygidae were set to 2 km based on expert opinion and because hand-wing index is not a useful predictor for flightless species. Values for *Diomedea exulans*, *Phoebastria immutabilis* and *Thalassarche chrysostoma* were based on published data, while those for the remaining species of Diomedeidae were set to the mean for these three species (4.909 km), as albatrosses are known to show high breeding philopatry despite the ability to travel large distances at sea.

To estimate dispersal distance in mammals we used the models in Whitmee & Orme (2012) and Santini et al. (2013) for mammals, depending on the trait data available (i.e. body mass, diet, home range area, weaning age and population density). Data on body mass and diet were retrieved from the EltonTraits database (Wilman et al. 2014), data on home range and weaning age from PanTHERIA database (Venter et al. 2016), and data on population density from the TetraDENSITY database (Santini et al. 2018a). As the minimum data requirements for these models were body mass and diet (Santini et al. 2013), we were able to predict dispersal distance for all species.

Table S3. Best model coefficient estimates.

	**Estimate**	**SE**	**t value**
Intercept	-3.14	0.58	-5.41
Hand Wing Index	1.62	0.33	4.95
Body Mass	0.39	0.10	3.96
Geographic Range	0.10	0.05	1.83

Fig. S3. Relationship between predicted dispersal distances and independent observed data obtained by the 5-fold cross-validation repeated 10 times.

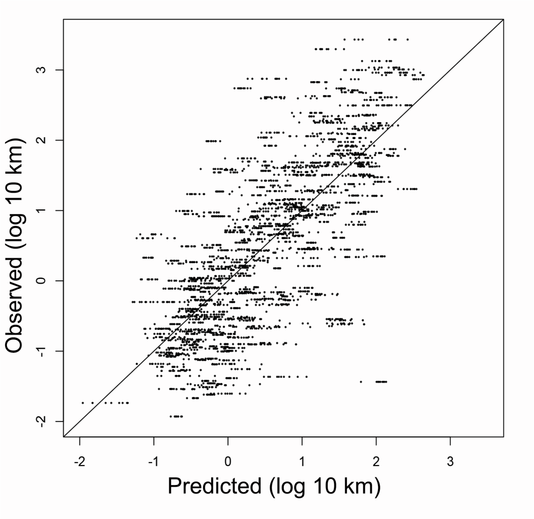

Appendix S2. Supplementary results

Table S4. Sensitivity of predictions to fragmentation criterion. NoFrag = No fragmentation criterion applied; 100km^2>50% = >50% of suitable area is in fragments smaller than <100km^2; *ind>50% = >50% of suitable habitat is in fragments supporting less than * individuals; V*>50% = >50% of suitable area is in fragments supporting less than a viability target in Hilbers *et al*. 2016, where * is the assumed proportion of maximum growth rate of the population; *"Category"=number of species predicted for that category; %Threatened= % of species predicted to be threatened; %Threatened_DD = % of species DD predicted to be threatened; %Under=Species predicted to be less threatened than under Red List; %Over=Species predicted to be more threatened than under Red List; nB1-2=times criterion B1-2 is applied (not necessarily determining the Red List category; other criteria can predict higher threats); Condition=times the fragmentation criterion is met.

Class	Fragmentation	nLC	nVU	nEN	nCR	%Threatened	%Threatened_DD	%Under	%Over	nB1	nB2	Condition	Sensitivity	Specificity	TSS	Gamma
Birds	NoFrag	9083	397	252	76	7.39	18.87	4.63	10.09	402	254	885	0.28	0.96	0.24	0.754 ***
	100km2>50%	9408	195	130	75	4.08	15.09	2.17	10.86	81	113	34	0.2	0.98	0.18	0.825 ***
	100ind>50%	9455	184	95	74	3.6	15.09	1.9	11.05	20	48	16	0.18	0.98	0.16	0.825 ***
	500ind>50%	9436	183	114	75	3.79	15.09	2.04	10.98	54	89	23	0.18	0.98	0.17	0.821 ***
	1000ind>50%	9414	191	128	75	4.02	16.98	2.18	10.91	78	114	30	0.19	0.98	0.17	0.82 ***
	5000ind>50%	9339	214	179	76	4.78	16.98	2.62	10.61	150	187	69	0.22	0.98	0.2	0.817 ***
Mammals	NoFrag	4260	266	206	29	10.52	22.31	3.37	20.31	367	236	3358	0.27	0.96	0.23	0.741 ***
	100km2>50%	4517	99	116	29	5.12	13.31	1.74	22.67	116	137	316	0.12	0.98	0.1	0.731 ***
	100ind>50%	4639	65	36	21	2.56	5.48	0.85	23.61	10	21	26	0.07	0.99	0.06	0.788 ***
	500ind>50%	4615	67	52	27	3.07	7.05	1.06	23.49	31	52	82	0.08	0.99	0.07	0.761 ***
	1000ind>50%	4603	70	61	27	3.32	7.63	1.18	23.4	42	66	116	0.08	0.99	0.07	0.75 ***
	5000ind>50%	4562	79	92	28	4.18	10.37	1.41	23.02	80	107	225	0.1	0.99	0.09	0.75 ***
	V0.2>50%	4491	119	122	29	5.67	14.68	1.84	22.46	139	152	405	0.13	0.98	0.12	0.727 ***
	V0.4>50%	4587	74	72	28	3.65	9.2	1.25	23.3	52	76	130	0.09	0.99	0.08	0.75 ***
	V0.6>50%	4612	68	54	27	3.13	7.44	1.08	23.47	30	51	75	0.08	0.99	0.07	0.754 ***
	V0.8>50%	4632	66	40	23	2.71	6.46	0.85	23.61	14	27	32	0.07	0.99	0.06	0.793 ***
	V1>50%	4639	65	34	23	2.56	5.87	0.85	23.73	6	16	19	0.07	0.99	0.06	0.788 ***

Fig. S4. Mean difference between published and predicted IUCN Red List categories by biogeographic realm, where LC/NT=0, VU=2, EN=3, CR=4. Category abbreviations match those in Fig. 2. Positive values indicate that predicted Red List categories were lower than the current published categories on the IUCN Red List. Error bars encompass the 90% of the distribution of the differences.

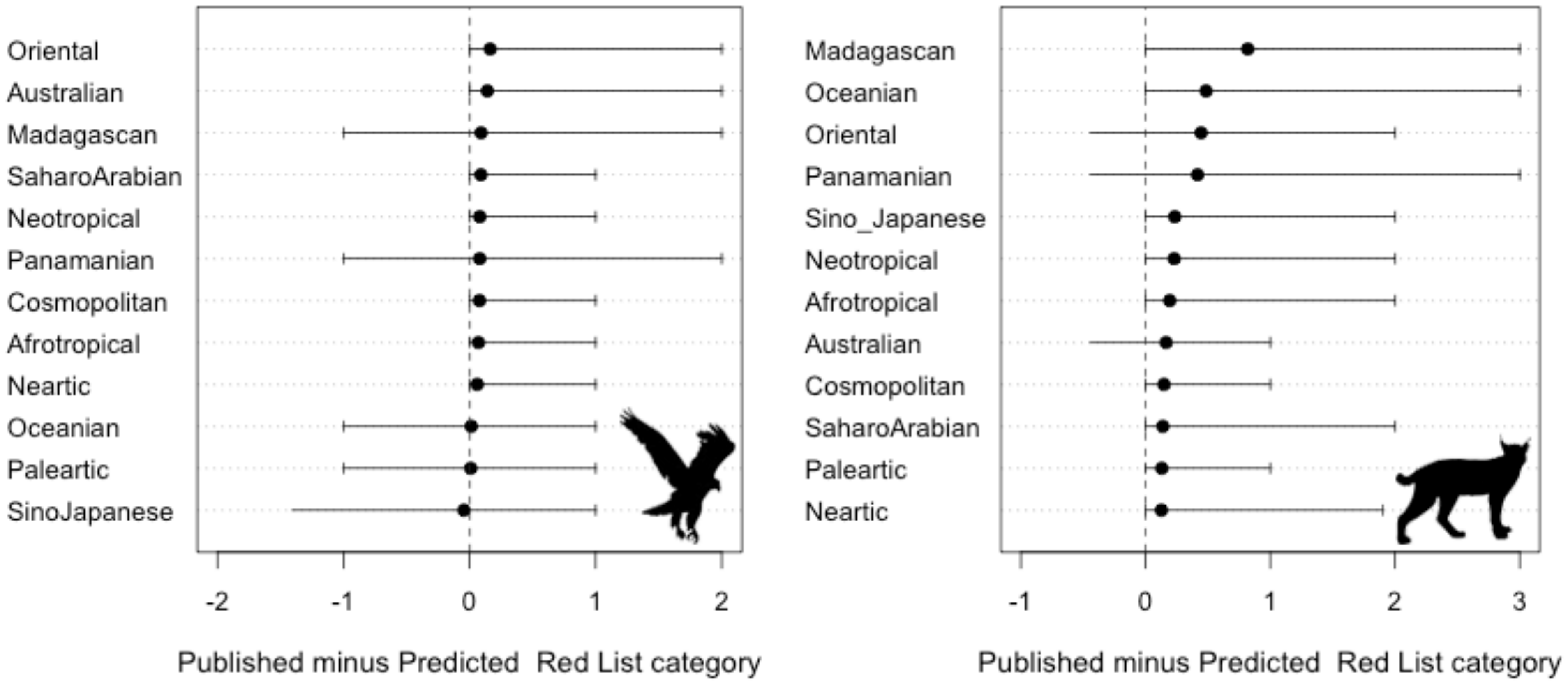

Fig. S5. Relationship between species body mass and difference between the observed and predicted Red List categories. Positive values indicate that the predicted threat category is lower than the observed category.

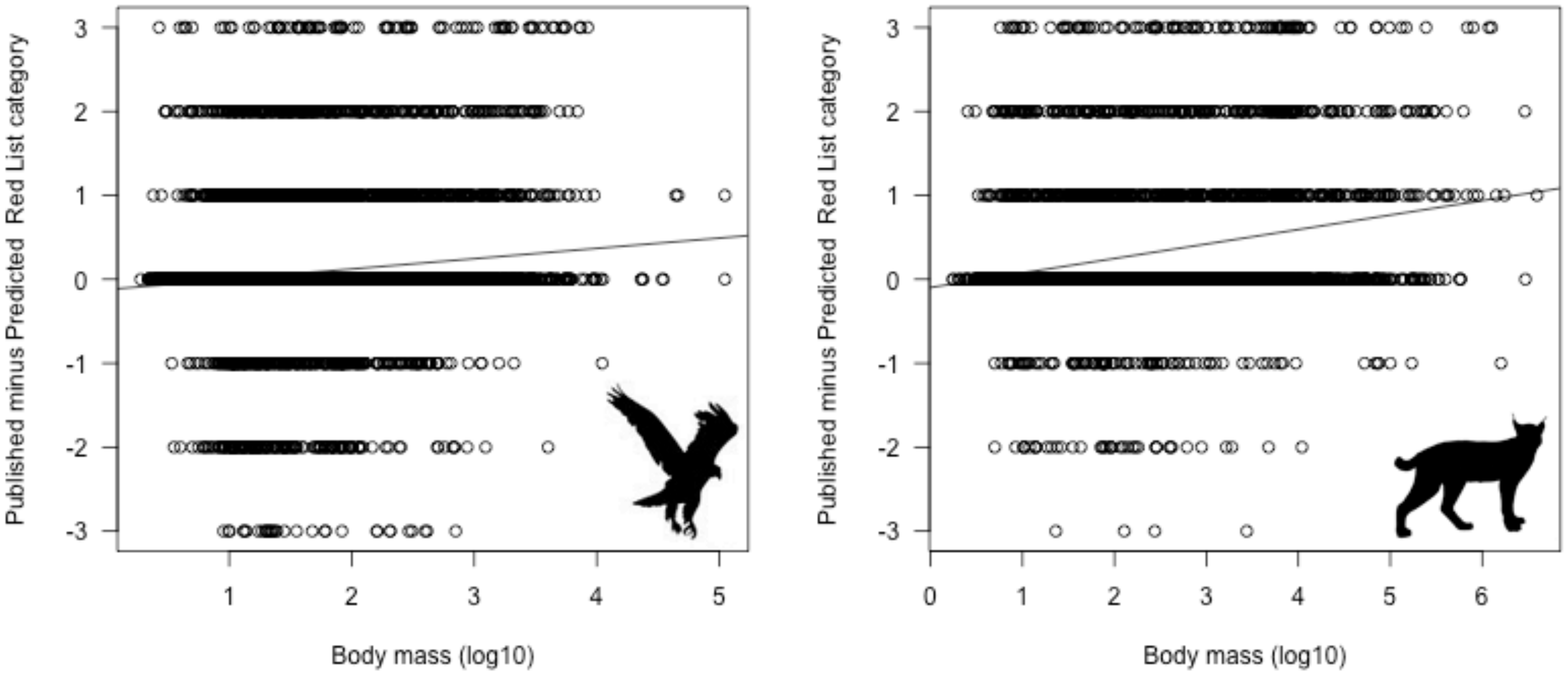

Appendix S2.1. Comparison between predicted and published Red List categories

We used the Goodman and Kruskal's Gamma statistics (Goodman & Kruskal 1954) to quantify the consistency between our predictions and the published Red List categories from the 2017 IUCN Red List. G-K Gamma measures the association between two discrete ordinal variables from contingency tables, and presents values ranging from -1 (perfect inverse association) to 1 (perfect positive association). We calculated also the sensitivity (rate of species correctly classified as threatened) and specificity (rate of species correctly classified as non-threatened) of our predictions to assess how well the approach is able to discriminate between threatened and non-threatened species. For this purpose, we converted our predicted Red List assessments to binary categories, non-threatened (Near Threatened and Least Concern) and threatened (Vulnerable, Endangered and Critically Endangered), following previous studies (e.g. Price & Gittleman 2007; Davidson et al. 2009). As a measure of overall accuracy, we used the True Skill Statistics (TSS) that is calculated as

$$TSS = Sensitivity + Specificity - 1$$

TSS ranges from -1 to 1, where negative values indicate a predictive ability worse than random, and positive values better than random (Allouche et al. 2006). These predictive accuracy measures implicitly assume that published Red List categories represent the “true” extinction risk category. While this assumption is acceptable for the scope of this analysis, we should be aware that published assessment are subject to uncertainty due to missing or biased knowledge. Therefore, while predictions are less likely to be accurate than published assessments, mismatches may also inform us about possible inaccurate assessments.

Finally, we investigated whether mismatches between predicted and published assessments vary across taxonomic orders, biogeographic regions (as defined in Holt et al. 2013), and in relation to species body mass. We measured the mismatch as the difference between two ordinal values (where LC and NT=0, VU=1, EN=2, CR=3). We note that extinction risk does not increase linearly

with increasing threat category (i.e. categories are not evenly spaced in relation to extinction risk). However, our approach is not intended to measure agreement between predicted and actual extinction risk, but rather to measure the extent to which predictions are distinct from published categories. We used body mass data from EltonTraits database (Wilman et al. 2014) and species' range polygons to estimate the mismatch by grid cell at 0.5 degree resolution.

Appendix. S2.2. Data Deficient species category estimation and comparison with different approaches

We compared our predictions for Data Deficient species with predictions produced in previous published works. For birds, we used expert judgements of the likely true category for each Data Deficient species published in Butchart and Bird (2010). For mammals we used predictions based on machine learning algorithms published in Bland *et al.* (2015). These were obtained by training several machine learning algorithms on assessed species (non-Data Deficient) that related species traits, geographic distribution and threat information with species conservation status, and then predicted the status of Data Deficient species. Our predictions showed agreement with those produced by expert judgements (Butchart & Bird 2010), whereas they were less consistent with those obtained using machine learning algorithms (Bland et al. 2015). In birds, predictions were concordant in 76.2% of the species but their correlation was low (K-G Gamma = 0.11; p-value = 0.90). In fact, predictions were mostly concordant for non-threatened species (73.8%) and only 1 species was consistently predicted as threatened (Letitia's thorntail, *Discosura letitiae*). In mammals the correlation was higher with K-G Gamma of 0.948 (p-value < 0.001) but predictions were concordant in 56.6% of the species, with 36.1% of species consistently predicted to be non-threatened, and 20.4% to be threatened. Overall, we predicted less threatened species than in Bland *et al.* (2015) (i.e. 90 *vs*. 185). Our approach mostly focuses on changes in habitat availability over time, while the approach in Bland *et al.* (2015) mostly focuses on species' intrinsic vulnerability to extinction. Therefore these approaches, as well as expert judgements, can be informative and their use can be complementary in identifying species that require urgent monitoring and targeted research.

Appendix S2.3. Sensitivity to suitable habitat and abundance estimates

To assess the extent to which our assumptions on habitat occupancy and population size influence our final predictions, we re-classified species by assuming decreasing proportions of suitable habitat and abundance, and all their possible combinations at 0.1 intervals. We then measured TSS for each of these combinations.

The highest G-K Gamma correlation value between our predictions and the published Red List assessments was obtained by assuming a population size equal to 10% of the total population size estimated based on modelled density, and an AOO from to 70% to 100% of the full AOO in birds (G-K Gamma=0.787) and mammals (G-K Gamma=0.790) respectively (Fig. S5). Further, the TSS was maximized by considering the AOO equal to 10% of the ESH and population size equal to 10% of our estimated total population size based on modelled density in both birds (TSS=0.406) and mammals (TSS=0.322). If we were using these assumptions as corrections for our predictions, the number of Data Deficient species would increase from 10 to 19 (maximized K-G Gamma) and 23 (maximized TSS) in birds, and from 114 to 145 (maximized K-G Gamma) and 187 (maximized TSS) in mammals (Fig. S6). Further, the number of species predicted to be more at risk than under the Red List would increase from 467 to 698 (maximized K-G Gamma) and 1093 (maximized TSS) in birds and from 143 to 322 (maximized K-G Gamma) 560 mammals (maximized TSS) in mammals (Fig. S6). This indicates that the number of threatened species might be much higher than currently predicted.

Fig. S6. Sensitivity of the True Skill Statistics (TSS) and Goodman and Kruskal's Gamma statistics (G-K Gamma) to decreasing proportions of population size and suitable habitat.

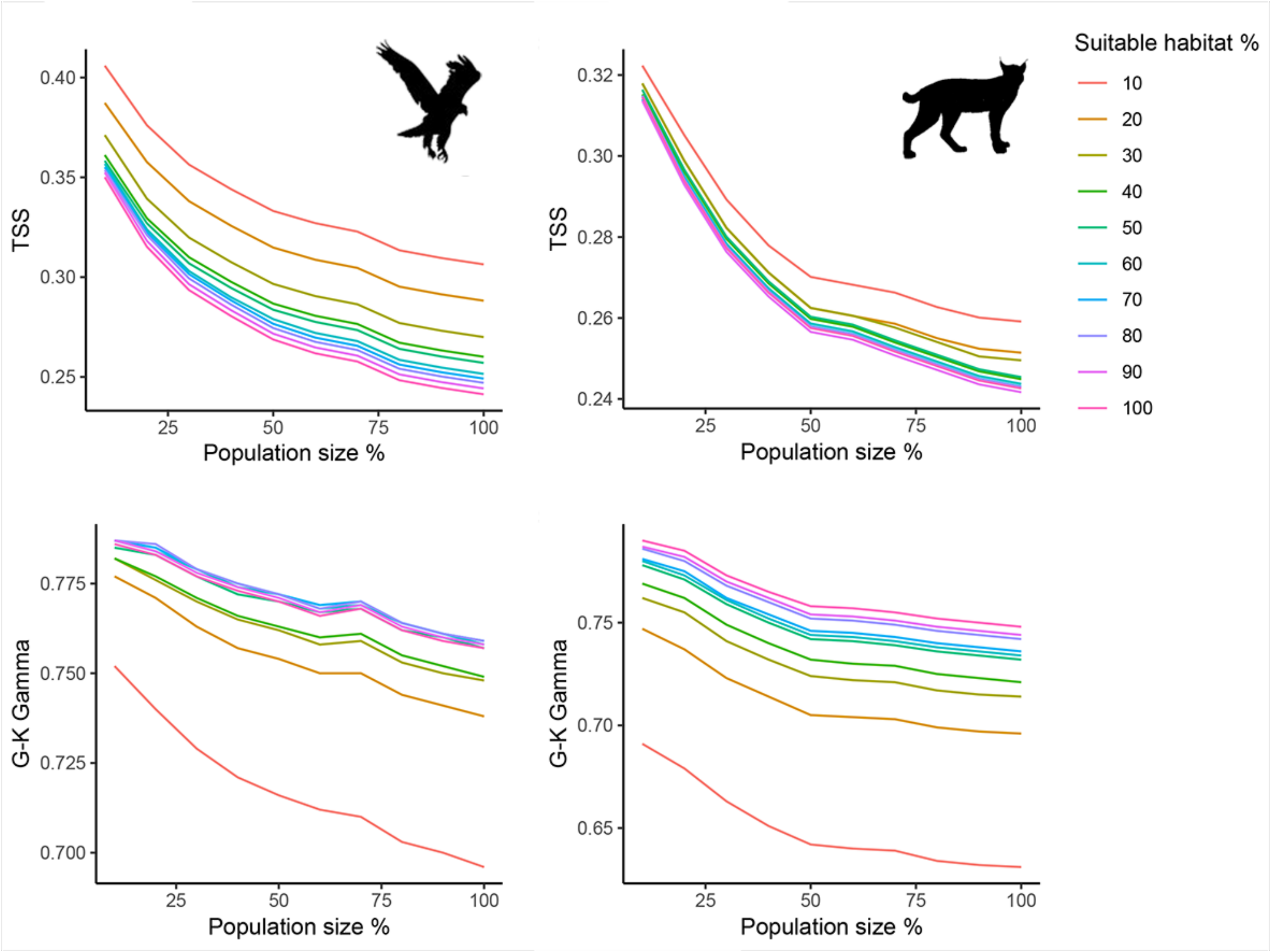

Fig. S7. Effect of decreasing proportions of population size and suitable habitat on the number of Data Deficient species predicted to be threatened, and the number of species predicted to be more threatened than currently classified under Red List criteria.

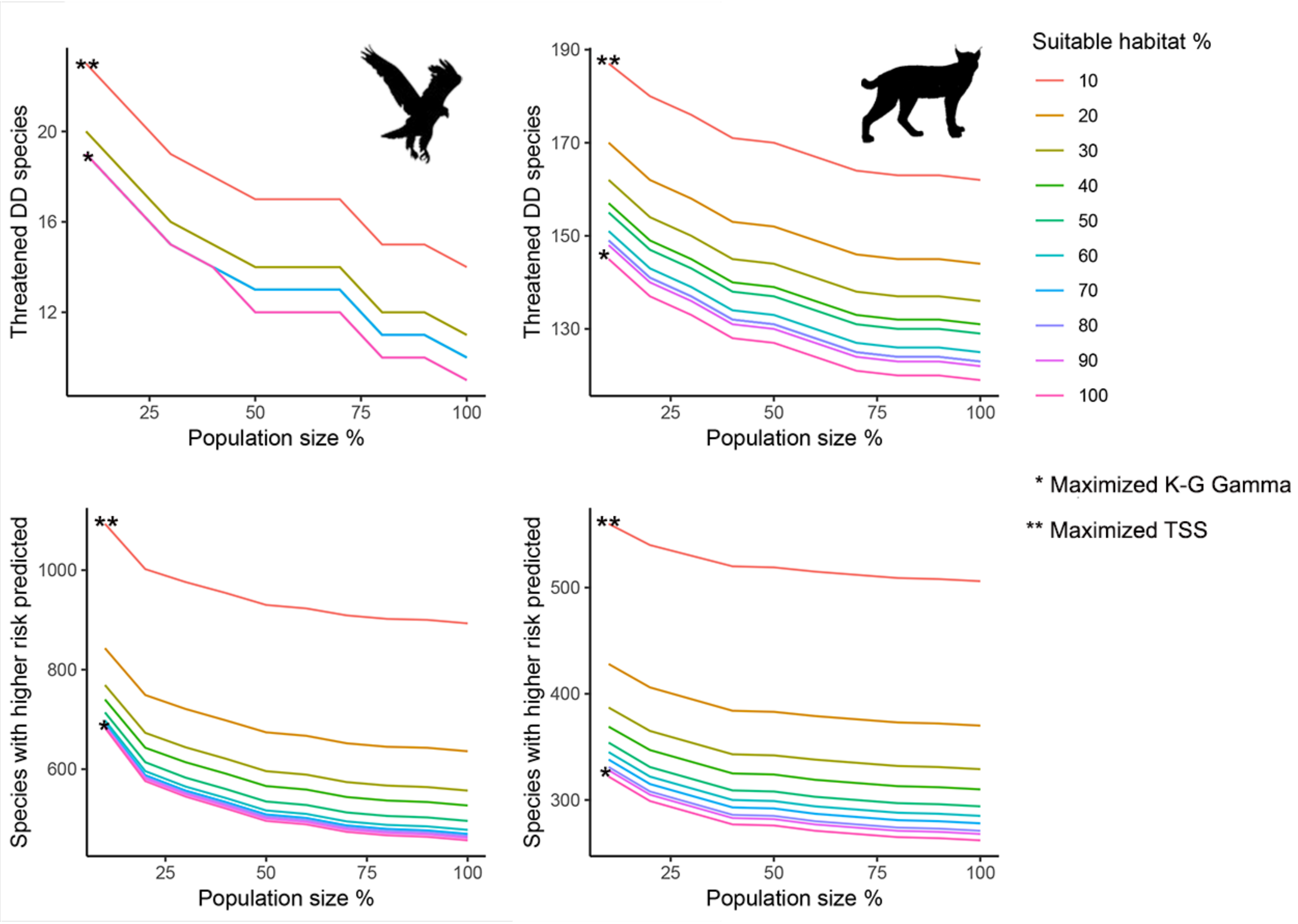

References

Allouche O, Tsoar A, Kadmon R. 2006. Assessing the accuracy of species distribution models: Prevalence, kappa and the true skill statistic (TSS). Journal of Applied Ecology.

BirdLife International. 2017. IUCN Red List for birds.

BirdLife International and Handbook of the Birds of the World. 2017. Bird species distribution maps of the world. Version 6.0. Available at http://datazone.birdlife.org/species/requestdis.

Bland LM, Collen B, Orme CDL, Bielby J. 2015. Predicting the conservation status of data-deficient species. Conservation Biology **29**:250–259.

Butchart SHM, Bird JP. 2010. Data Deficient birds on the IUCN Red List: What don't we know and why does it matter? Biological Conservation **143**:239–247.

Claramunt S, Derryberry EP, Remsen J V., Brumfield RT. 2012. High dispersal ability inhibits speciation in a continental radiation of passerine birds. Proceedings of the Royal Society B: Biological Sciences **279**:1567–1574. Available from http://rspb.royalsocietypublishing.org/cgi/doi/10.1098/rspb.2011.1922.

Davidson AD, Hamilton MJ, Boyer AG, Brown JH, Ceballos G. 2009. Multiple ecological pathways to extinction in mammals. Proceedings of the National Academy of Sciences, USA **106**:10702–10705.

Dawideit BA, Phillimore AB, Laube I, Leisler B, Böhning-Gaese K. 2009. Ecomorphological predictors of natal dispersal distances in birds. Journal of Animal Ecology **78**:388–395.

Goodman LA, Kruskal WH. 1954. Measures of Association for Cross Classifications. Journal of the American Statistical Association **49**:732–764.

Hijmans RJ, Cameron SE, Parra JL, Jones PG, Jarvis A. 2005. Very high resolution interpolated climate surfaces for global land areas. International Journal of Climatology **25**:1965–1978.

Holt BG et al. 2013. An Update of Wallace's Zoogeographic Regions of the World. Science **339**:74–78.

Imhoff M, Bounoua L, Ricketts T, Loucks C, Harriss R, Lawrence WT. 2004. Global patterns in

human consumption of net primary production. Nature **429**:870–873. Available from http://www.nature.com/nature/journal/v429/n6994/abs/nature02619.html.

IUCN. 2017. Guidelines for using the IUCN Red List Categories and Criteria. Version 13. Prepared by the Standards and Petition Subcommittee. Downloadable from http://www.iucnredlist.org/documents/RedListGuidelines.pdf.

Kennedy JD, Borregaard MK, Jønsson KA, Marki PZ, Fjeldså J, Rahbek C. 2016. The influence of wing morphology upon the dispersal, geographical distributions and diversification of the Corvides (Aves; Passeriformes). Proceedings of the Royal Society B: Biological Sciences **283**:20161922. Available from http://rspb.royalsocietypublishing.org/lookup/doi/10.1098/rspb.2016.1922.

Pigot AL, Tobias JA. 2014. Dispersal and the transition to sympatry in vertebrates. Proceedings of the Royal Society B: Biological Sciences **282**:20141929–20141929. Available from http://rspb.royalsocietypublishing.org/cgi/doi/10.1098/rspb.2014.1929.

Pigot AL, Trisos CH, Tobias JA. 2016. Functional traits reveal the expansion and packing of ecological niche space underlying an elevational diversity gradient in passerine birds. Proceedings of the Royal Society B: Biological Sciences **283**:20152013. Available from http://rspb.royalsocietypublishing.org/lookup/doi/10.1098/rspb.2015.2013.

Price SA, Gittleman JL. 2007. Hunting to extinction: Biology and regional economy influence extinction risk and the impact of hunting in artiodactyls. Proceedings of the Royal Society B: Biological Sciences **274**:1845–1851.

Santini L, Di Marco M, Boitani L, Maiorano L, Rondinini C. 2014. Incorporating spatial population structure in gap analysis reveals inequitable assessments of species protection. Diversity and Distributions **20**:698–707.

Santini L, Di Marco M, Visconti P, Daniele B, Luigi B, Rondinini C. 2013. Ecological correlates of dispersal distance in terrestrial mammals. Hystrix, The Italian Journal of Mammalogy **24**:181–186.

Santini L, Isaac NJB, Ficetola GF. 2018a. TetraDENSITY: a database of population density estimates in terrestrial vertebrates. Global Ecology & Biogeography **27**:787–791.

Santini L, Isaac NJB, Maiorano L, Ficetola GF, Huijbregts MAJ, Carbone C, Thuiller W. 2018b. Global drivers of population density in terrestrial vertebrates. Global Ecology & Biogeography **27**:968–979.

Stoddard MC, Yong EH, Akkaynak D, Sheard C, Tobias JA, Mahadevan L. 2017. Avian egg shape: Form, function, and evolution. Science **356**:1249–1254.

Sutherland GD, Harestad AS, Price K, Lertzman KP. 2000. Scaling of natal dispersal distances in terrestrial birds and mammals. Conservation Ecology **4**:16.

Venter O et al. 2016. Sixteen years of change in the global terrestrial human footprint and implications for biodiversity conservation. Nature Communications **7**:11.

Whitmee S, Orme CDL. 2012. Predicting dispersal distance in mammals: a trait-based approach. Journal of Animal Ecology **82**:211–221.

Wilman H, Belmaker J, Simpson J, de la Rosa C, Rivadeneira MM, Jetz W. 2014. EltonTraits 1.0: Species-level foraging attributes of the world's birds and mammals. Ecology **95**:2027–2027. Available from http://www.esajournals.org/doi/abs/10.1890/13-1917.1.
